# Immediate Oncoplastic Breast Reconstruction with Fat Grafting: Preliminary Radiological, Aesthetic, and Patient Satisfaction Outcomes

**DOI:** 10.1007/s00266-025-04790-3

**Published:** 2025-04-07

**Authors:** Lior Har-Shai, Tomer Lagziel, Ahuva Grubstein, Tamir Shay, Dean Ad-El, Sagit Meshulam-Derazon, Eran Sharon, Michael Icekson

**Affiliations:** 1 Department of Plastic Surgery and Burns, Rabin Medical Center, Beilinson Hospital, Petah Tikva, Israel; 2 Department of Imaging, Rabin Medical Center, Beilinson Hospital, Petach Tikva, Israel; 3 Department of Breast Surgery, Rabin Medical Center, Beilinson Hospital, Petah Tikva, Israel; 4 Faculty of Medicine, Tel-Aviv University, Tel-Aviv, Israel; 5 Department of Surgery, Icahn School of Medicine at Mount Sinai, New-York, NY USA

**Keywords:** Immediate fat grafting, Oncoplastic reconstruction, Breast reconstruction, Breast cancer

## Abstract

**Introduction:**

Fat grafting is a valuable tool for oncologic breast reconstruction, as it enhances aesthetic outcomes. However, concerns regarding oncologic safety and challenges in postoperative imaging have limited the adoption of immediate oncoplastic breast reconstruction (IOBR) with fat grafting. This approach can reduce the need for additional surgeries, shorten recovery time, improve aesthetics, and help mitigate the adverse effects of adjuvant radiation therapy. This study evaluates postoperative, radiological, aesthetic, and patient-reported outcomes of immediate fat grafting in oncoplastic breast reconstruction following lumpectomy.

**Methods:**

We conducted a retrospective study of patients undergoing IOBR with immediate fat grafting following lumpectomy (2020–2022). The plastic surgery team performed reconstruction simultaneously with lumpectomy by breast surgeons. Patient satisfaction was assessed using the Breast-Q questionnaire, while expert surgeons evaluated aesthetic outcomes. Lesion characteristics, specimen weight, and postoperative radiation details were recorded. Postoperative breast imaging was reviewed for fat grafting-related abnormalities.

**Results:**

Fifteen patients were included, with 87% undergoing postoperative radiotherapy. No major complications or readmissions occurred within 30 days. Breast imaging follow-up showed 91.1% had benign post-surgical changes, while 8.9% required short-term radiologic follow-up. Post-lipoid injection findings appeared in 37.8% of cases, none with calcifications. Patient satisfaction was high (average Breast-Q Score was 74.5), with only one patient requesting additional fat grafting post-radiation. Expert assessments confirmed improved aesthetic outcomes.

**Conclusion:**

IOBR with immediate fat grafting is a useful technique for lumpectomy defects across all breast quadrants, demonstrating low complication rates, high patient satisfaction, and positive aesthetic outcomes. Postoperative imaging follow-up revealed no adverse effects related to fat grafting, supporting its potential role as an additional tool in oncoplastic breast reconstruction.

**Level of Evidence IV:**

This journal requires that authors assign a level of evidence to each article. For a full description of these Evidence-Based Medicine ratings, please refer to the Table of Contents or the online Instructions to Authors www.springer.com/00266.

**Supplementary Information:**

The online version contains supplementary material available at 10.1007/s00266-025-04790-3.

## Introduction

Breast cancer affects millions of women globally, with 2.3 million cases diagnosed in 2020, making it the most common cancer among women [1]. Treatment has evolved from radical mastectomy to more conservative approaches like breast-conserving therapy (BCT) [2, 3].

BCT, combining lumpectomy or quadrantectomy with radiotherapy, has become the standard of care for early-stage breast cancer, ensuring oncologic safety while reducing physical and psychological burdens. [4–7]

Although BCT often yields satisfactory outcomes for small lumpectomies, significant glandular tissue removal can lead to aesthetic deformities and breast asymmetry, impacting body image, psychological well-being, and quality of life. This highlights the need for effective reconstructive options. [8]

Reconstruction after BCT encompasses contralateral symmetry procedures, local tissue rearrangements, regional flaps and simple closures [9, 10]. Recently, oncoplastic surgery, which integrates oncologic and plastic surgery principles, has gained traction [11]. Notably, delayed autologous fat grafting (AFG) has shown promise in mitigating the effects of radiation therapy while enhancing breast symmetry and contour post-lumpectomy. [12, 13]

Controversy remains over the long-term effects of AFG, particularly its potential impact on cancer recurrence due to stem cell-containing injections at a former tumor site. However, growing evidence supports AFG’s safety in breast reconstruction for both mastectomy and BCT patients. Studies, including those on postmastectomy cases, show no increased recurrence risk [14–18]. Notably, Krastev et al. [19] found no significant difference in locoregional recurrence between AFG and control patients over a 5-year follow-up.

Recent studies have introduced *immediate* AFG for BCT patients, aiming to reconstruct lumpectomy or quadrantectomy defects while ensuring lasting aesthetic outcomes post-radiotherapy. Though limited, existing research shows comparable oncologic results to BCT without AFG as well as favorable aesthetic outcomes, highlighting the need for further studies [20–24]. A key concern is its impact on clinical and radiological follow-up, as fat necrosis can cause lumps and calcifications, complicating postoperative monitoring [25]. Addressing these challenges is essential in advancing breast reconstruction techniques.

This study aimed to share our experience with immediate oncoplastic breast reconstruction (IOBR) using fat grafting as a volume replacement technique for BCT patients, assessing its outcomes with a focus on complications, patient satisfaction, and postoperative radiologic findings. By contributing to the growing literature on this novel approach, it provides valuable insights into its clinical application.

## Methods

### Patient Selection

#### Inclusion Criteria

- Patients with benign or malignant breast tumors who were scheduled for lumpectomy and expressed and interest in oncoplastic breast reconstruction.
- Sufficient donor site availability for fat grafting.

#### Exclusion Criteria

- Patients eligible for volume displacement oncoplastic procedures (e.g., breast reduction or mastopexy) based on breast shape, volume, or degree of ptosis.
- Active smokers.
- Previous breast irradiation

### Technique

Our surgical technique for IOBR with lipofilling begins with preoperative marking, while the patient is in a standing position. Key landmarks such as the midline, inframammary folds, bilateral breast footprint, the planned location of the breast defect, and the donor sites for fat harvesting are marked. The breast incision site is also carefully chosen collaboratively with the breast surgeon to provide optimal access to the tumor with minimal visible scarring.

A tumescent solution is prepared combining 400mg of lidocaine with 2mg of adrenaline in 1 liter of Hartmann solution. This solution is injected into the donor sites to minimize bleeding and facilitate fat harvesting (Video 1). The size of the breast defect and tissue deficit post-lumpectomy is determined and recorded. Hemostasis is achieved to control bleeding from the surgical site using cautery and ligation, as necessary.

Based on the size and location of the breast defect, local tissue flaps are dissected and transferred to close the defect. These flaps can be advanced or rotated as needed to ensure complete coverage of the defect and avoid dead space (Video 2). Vicryl 2-0 sutures are used to secure the flaps in place.

Fifteen minutes following the tumescence injection, manual liposuction is performed at the donor sites, using a Luer-lock syringe (10cc) and a Coleman aspiration cannula (2mm). Approximately 120% of the lumpectomy specimen weight is aspirated in volume. The syringes with the extracted fat are placed vertically to facilitate the layer separation and division of the lipoaspirate. The oil and aqueous layers are then discarded. The prepared fat is transferred to 5cc syringes via a 3-way stopcock with 2 female Luer locks and tubing port.

The processed fat is subsequently grafted using an infiltration technique to fill the tissue around the donor sites of the local tissue flaps and correct secondary contour defects (Video 3). Fat grafting is performed in three dimensions, focusing on the deep plane adjacent to the pectoralis muscle, the breast parenchyma, and subcutaneous tissue. Most of the fat is grafted while the skin incision is still open though all other tissues are in place. A small amount is reserved for finesse grafting after the skin is closed. Fat equalization is performed in the donor sites to ensure a smooth contour.

### Data Collection

After approval of the institutional IRB committee (RMC-0372-23), we queried our electronic records for all patients with pre-existing breast masses who underwent BCT with immediate fat grafting at our institution from 2020 to 2022. The data points collected for each patient included preoperative, operative, and postoperative variables.

Preoperative data included demographics, BMI, past medical history (PMH), smoking status, breast cup size, previous radiotherapy, previous breast disease, and management.

Operative data included type and grade of cancer, receptor status, node status, type of breast surgery, type of reconstruction, specimen weight, pathologic quadrant, fat graft volume, and donor site.

Postoperative data included complications, follow-up evaluations, details of radiation treatment (number of cycles and total radiation dose), 30-day readmission, reoperation plans, Breast-Q Scores (Reconstruction Module (Postoperative) Version 2.0: Satisfaction with Breasts), and validated aesthetic assessments independently scored by three board-certified plastic surgeons.

Additionally, all patients underwent postoperative imaging studies in accordance with the American College of Radiology appropriateness criteria. Imaging included annual mammographic surveillance, with the first post-treatment mammogram performed six months after therapy completion. Adjunct ultrasound and MRI were used in diagnostic cases and for patients at elevated risk [26]. All postoperative imaging studies were reviewed by a breast radiologist and assessed using the Breast Imaging Reporting and Data System (BI-RADS) classification [26, 27]. The analysis included a detailed evaluation of imaging findings related to fat grafting. Typical findings, such as well-demarcated calcifications, oil cysts, and scar-related architectural distortions on mammography and ultrasound, as well as fat signal within a lesion with or without enhancement on MRI, were interpreted as post-surgical and fat grafting changes [28].

## Results

Between 2020 and 2022, 15 patients diagnosed with breast lesions necessitating lumpectomies underwent IOBR with immediate fat grafting (Table 1). The mean patient age was 54 years.

**Table 1:** Demographics and preoperative data (*n *= 15)

Age (years)	Height (cm)	Weight (kg)	BMI (Kg/m^2^)	Comorbidities	Smoking	Preoperative breast cup size
53.9 +/− 12.7	164.6 +/− 6.2	67.9 +/− 8.2	25.08 +/− 3.1	46.7%	None	B=40% C=60%

All patients underwent lumpectomies, and the average mass of the excised tissue was 49.2gr, while the mean volume of fat grafted during the operation was 66.8cc. The hospital length of stay was 2.1 days; no patients stayed longer than 3 days total (Table 2). The average time from surgery to follow-up was 16.5 (range: 6–25) months. The average amount of time from the last radiation therapy to follow-up was 10.5 (range: 3–18) months. No locoregional recurrence of breast cancer was evident for all the patients included in this study.

**Table 2:** Intra-operative findings (*n *= 15)

Cancer type	Grade	Receptor status	Specimen weight (g)	Pathologic quadrant	Fat graft volume (cm^3^)	Admission length (days)
IDC = 46.6% ILC = 6.7% IDC+DCIS = 40% Fibroadenoma = 6.7%	1 = 13.3% 2 = 53% 3 = 20%	ER+, PR+ = 46.6% ER+, HR+ = 6.7% PR+ = 13.3% HR+ = 6.7% Triple positive = 13.3% Triple negative = 6.7%	49.2 +/− 25.1	LLLQ = 13.3% LLUQ = 20% RLUQ = 33.3% RLLQ = 6.7% RMUQ = 6.7% LC = 6.7% Multiple = 13.3%	66.8 +/− 36.9	2.1 +/− 0.4

IDC = Invasive ductal carcinoma, ILC = Invasive lobular carcinoma, DCIS = Ductal carcinoma in situ, ER**+** = Estrogen receptor positive, PR**+** = Progesterone receptor positive, HR**+** = Hormone receptor positive, LLLQ = Left lower lateral quadrant, LLUQ = Left upper lateral quadrant, RLUQ = Right upper lateral quadrant, RLLQ = Right lower lateral quadrant, RMUQ = Right medial upper quadrant, LC = Left central

Results showed that IOBR with immediate fat grafting was well tolerated by all patients and led to overall very positive outcomes (Table 3).

**Table 3:** Postoperative data (n=15)

Complications	30-Day readmission	Reoperation	Breast Q score	Aesthetic assessment	Postoperative radiation (Gy, Rounds)
6.7%	0	0	3.2 +/− 0.5	4.6 +/− 0.5	2.9 +/− 0.3, 15.3 +/− 0.6

**Fig. 1:**
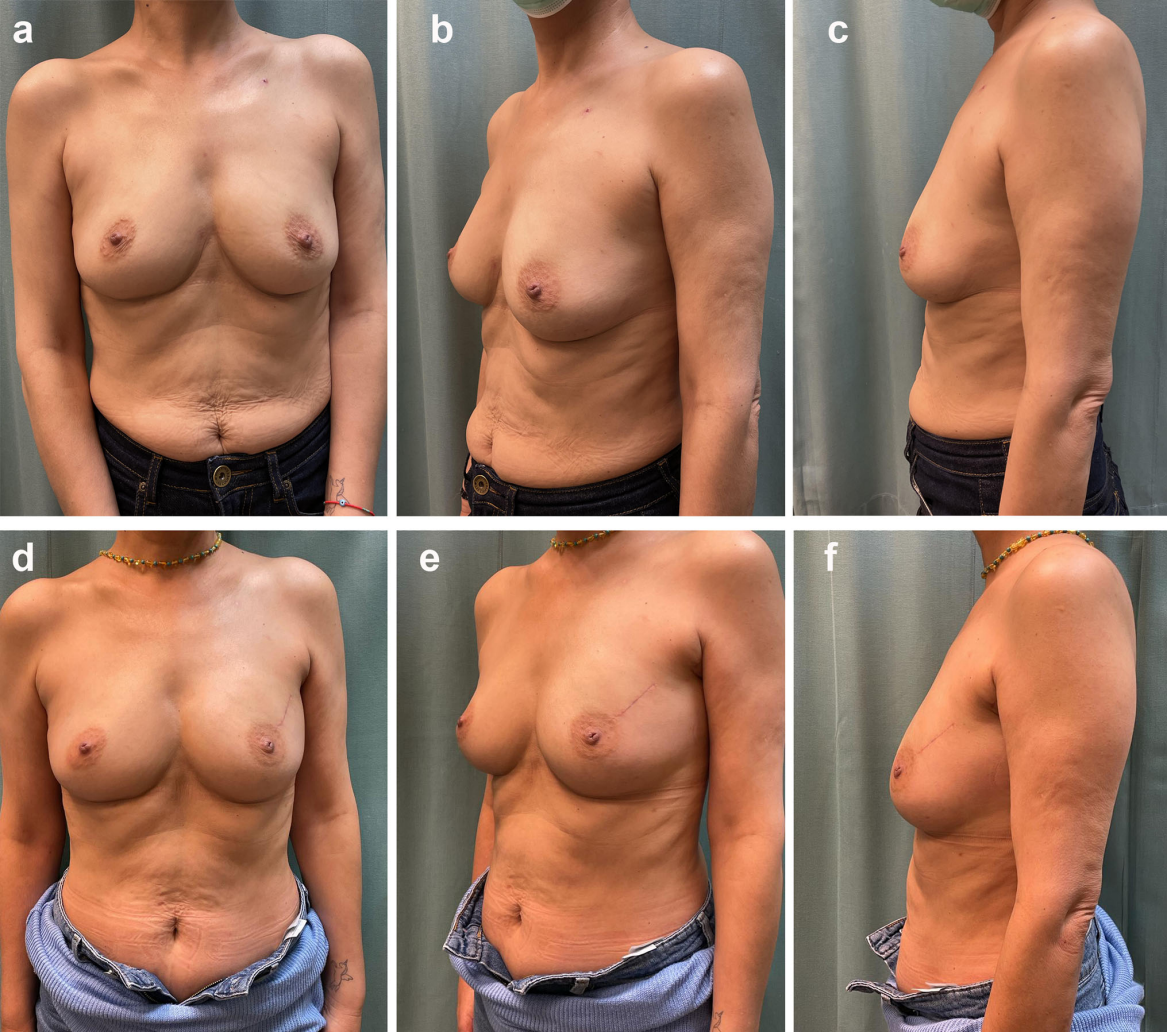
A 56-year-old woman, following neoadjuvant chemo-biological therapy, underwent a left breast lumpectomy in the supero-lateral quadrant, accompanied by IOBR with fat grafting. The lumpectomy specimen measured 7.4x5.3x2.1 cm and weighed 42 grams. 60cc of fat obtained from lateral thighs was grafted into the defect. Subsequently, the patient underwent radiotherapy comprising 15 sessions with a total dose of 40.05 Gy. The figure illustrates preoperative images of the patient (**a**–**c**) and the outcome 8 months after the completion of radiation therapy (**d**–**f**)

Postoperative Breast-Q scores, an established patient-reported outcome measure for breast surgery patients, also mirrored this satisfaction. Using a scale of 1–4, patients rated their postoperative satisfaction based on different pre-determined categories. The scores were then normalized and adjusted based on validated criteria. Based on these criteria, the average Breast-Q score was 74.5, on follow-up (Fig. 1).

For surgeon-centered aesthetic assessment, we used the Validated Breast Aesthetic Scale [29]. This assessment uses a 1–5 scale for rating different aesthetic portions of the reconstructed breast: breast, scar, nipple-areolar-complex (NAC), and overall appearance. Three board-certified plastic surgeons independently examined the patients and provided their scores. The average score for our cohort was 4.6.

As shown in Table 3, there was one postoperative complication, a single case of fat necrosis. No major complications or readmissions were noted within the 30-day postoperative period. Only one patient expressed interest in an additional procedure for aesthetic enhancement, but upon surgeon assessment, reoperation was not deemed necessary. A total of 13/15 patients underwent postoperative radiotherapy at an average of 15.3 sessions per patient with an average of 2.9 gray units of radiation per session.

As summarized in Table 4, out of 45 breast imaging studies, including MRI, ultrasound (US), and mammography, performed between 11 and 28 months following the surgical procedure, only 4 out of 45 (8.9%) revealed findings requiring short-term follow-up (BIRADS-3). Remarkably, in 3 of these cases (6.7%), the identified findings were directly associated with the surgical procedure itself. In a significant majority of the cases, 41/45 (91.1%), the surgical site was reported as having benign post-surgical changes (BIRADS-2). Findings related to post-lipoid injection were noted in 17 out of 45 (37.8%) studies, with all but three cases concluding these findings to be typically benign. In these exceptional instances, breast MRI detected fat necrosis enhancement. Additional typical changes included the presence of oil cysts, seroma or fat/fluid within the scar, and oil cysts with a mass. None of those changes calcified.

**Table 4:** Postoperative imaging findings (*n *= 15)*

	Total *n *= 45	
Breast Imaging Type, *n*(%)		
Mammography	17 (37.8)	
Ultrasound	17 (37.8)	
MRI	11 (24.4)	
Follow-up Length, months (mean)	11–28 (18)	
BIRADS Classification, n(%)		
BIRADS-2^**^	41 (91.1)	
BIRADS-3^***^	4 (8.9) [of them 3 were fat grafting related]	
Imaging findings associated with fat grafting, n(%)	*n *= 17 (37.8)	Relevant findings BIRADS
Oil cysts	8 (47.1)	2
Mass and oil cysts	1 (5.9)	2
Seroma of fat graft in scar	5 (29.4)	2
Fat necrosis enhancement	3 (17.6)	3
Calcifications	0	

BIRADS = Breast imaging reporting and data system

^*^This “*n*” references the total patients studied

^**^BIRADS-2: Benign findings

^***^BIRADS-3: Probably benign findings, 6-month follow-up is recommended

## Discussion

While exact reports vary, some studies report that nearly 40% of women undergoing BCT opt for oncoplastic reconstruction [30]. The decision to seek reconstructive surgery is multifactorial and influenced by factors such as the perceived impact of breast appearance on quality of life, body image, and sexual well-being. Our study suggests that IOBR with immediate fat grafting post-lumpectomy is a useful technique that can yield satisfactory results in terms of both aesthetic outcomes and patient satisfaction.

Emerging evidence suggests a potential radioprotective mechanism associated with fat grafting. Fat grafting to the breast has been hypothesized to stimulate a healing response, possibly improving the quality and vascularization of irradiated tissues. There are multiple theoretical contributing pathways. [12, 13, 31].

Adipose tissue, from which the fat graft is derived, contains a significant population of mesenchymal stem cells (MSCs) [32, 33]. MSCs have the potential to differentiate into multiple cell types, contributing to tissue repair in the context of irradiated tissue [34].

Furthermore, MSCs and other cell types in the fat graft secrete a range of growth factors, promoting angiogenesis and improving the vascularization of irradiated tissues [35, 36]. Improved blood flow can enhance oxygenation and nutrient supply to damaged tissues, aiding in their repair and recovery.

Additionally, fat grafting appears to exert immunomodulatory effects, potentially reducing inflammation in treated areas [37, 38]. Given that inflammation is a typical consequence of radiotherapy and can contribute to fibrosis and tissue damage, pre-radiation lipofilling might reduce the severity of radiation-induced complications.

Chronic fibrosis is a well-documented sequela of radiotherapy. The introduction of fat, accompanied by growth factors, may reverse or reduce the progression of fibrotic changes.

These mechanisms provide a plausible explanation for the observed benefits of immediate lipofilling. However, while promising, the radio-protective qualities of lipofilling require further validation in comprehensive studies and a longer follow-up.

IOBR with immediate fat grafting may offer several advantages over traditional reconstructive approaches. It offers a promising and useful tool for small to medium-sized lumpectomies situated in all breast quadrants. Importantly, it minimizes unnecessary manipulation of the healthy breast and the need of future contralateral procedures. Moreover, this technique circumvents potential complications associated with synthetic materials, crucial since many lumpectomy patients are likely to receive adjuvant radiotherapy. In addition, this approach seems to decrease the likelihood of future revision surgeries.

While our study presents promising results for IOBR with immediate fat grafting, it is important to weigh these benefits against the potential challenges in postoperative monitoring. The appearance of new lumps or calcifications can complicate the oncological follow-up, making it essential for clinicians to be aware of these potential outcomes and to educate patients accordingly. Additionally, it is essential to note that breast abnormalities and calcifications can occur with breast-conserving therapy (BCT) alone, as well as following other breast surgeries, such as breast reduction.

While post-fat-grafting changes were observed in some of the imaging studies we reviewed, they were either non-visible on the background of the lumpectomy scar, or typical benign post-fat grafting changes. This is in accord with the literature, where though higher utilization of diagnostic imaging due to the development of palpable lumps related to fat necrosis can be expected, in the majority of cases the imaging findings are typical and do not cause a diagnostic dilemma [39].

Despite its strengths, our study possesses several limitations. Its retrospective nature combined with a relatively small sample size limits the generalizability of our findings. Even when using standardized scales, aesthetic outcome assessments inherently contain an element of subjectivity. Furthermore, our follow-up duration is limited, which may not fully capture the potential protective effects of radiation or the long-term cosmetic outcomes.

To our knowledge, this is the first study to assess patient satisfaction following immediate oncoplastic breast reconstruction with fat grafting using Breast-Q scores.

Future research directions could include larger randomized controlled studies to robustly compare IOBR with immediate fat grafting against other reconstructive techniques. Implementing objective assessment tools like 3D imaging can further validate results. Additionally, long-term follow-up data would offer insights into the sustainability of aesthetic outcomes and patient satisfaction.

## Conclusions

Our study supports the use of IOBR with immediate fat grafting as a useful technique for breast reconstruction following lumpectomy across all breast quadrants, achieving high patient and surgeon satisfaction with aesthetic outcomes. This technique allows for simultaneous restoration of breast volume and shape at the time of lumpectomy, minimizing the need for additional surgeries and reducing recovery time. Consequently, it can help alleviate both the emotional and financial burdens on patients, making it a valuable option in breast-conserving therapy. Further studies with larger cohorts and long-term follow-up are needed to strengthen these conclusions and assess the durability of aesthetic and oncologic outcomes.

## Supplementary Information

Below is the link to the electronic supplementary material.

Supplementary file1 (MP4 92925 KB)

Supplementary file2 (MP4 60069 KB)

Supplementary file3 (MP4 76934 KB)

## Abbreviations

IOBR: Immediate oncoplastic breast reconstruction
BCT: Breast-conserving therapy
